# Pudexacianinium (ASP5354) chloride for ureter visualization in participants undergoing laparoscopic, minimally invasive colorectal surgery

**DOI:** 10.1007/s00464-023-10193-9

**Published:** 2023-07-20

**Authors:** Matthew Albert, Leticia Delgado-Herrera, Jennifer Paruch, Pauline Gerritsen-van Schieveen, Tomoyoshi Kishimoto, Shin Takusagawa, Na Cai, John Fengler, Jeffrey Raizer

**Affiliations:** 1 Department of Colorectal Surgery, Advent Health, 2415 N Orange Ave Ste 300, Orlando, FL 32804 USA; 2 Astellas Pharma Global Development, Inc., Northbrook, IL USA; 3 Ochsner Health, New Orleans, LA USA; 4 Astellas Pharma Europe B.V., Leiden, The Netherlands; 5 Astellas Pharma, Inc., Tokyo, Japan; 6 Stryker, Burnaby, BC Canada

**Keywords:** Indocyanine green, Colorectal surgery, Laparoscopy, Pudexacianinium, Fluorescent dyes, Near-infrared fluorescence

## Abstract

**Background:**

Intraoperative ureteral injury, a serious complication of abdominopelvic surgeries, can be avoided through ureter visualization. Near-infrared fluorescence imaging offers real-time anatomical visualization of ureters during surgery. Pudexacianinium (ASP5354) chloride is an indocyanine green derivative under investigation for intraoperative ureter visualization during colorectal or gynecologic surgery in adult and pediatric patients.

**Methods:**

In this phase 2 study (NCT04238481), adults undergoing laparoscopic colorectal surgery were randomized to receive one intravenous dose of pudexacianinium 0.3 mg, 1.0 mg, or 3.0 mg. The primary endpoint was successful intraoperative ureter visualization, defined as observation of ureter fluorescence 30 min after pudexacianinium administration and at end of surgery. Safety and pharmacokinetics were also assessed.

**Results:**

Participants received pudexacianinium 0.3 mg (*n* = 3), 1.0 mg (*n* = 6), or 3.0 mg (*n* = 3). Most participants were female (*n* = 10; 83.3%); median age was 54 years (range 24–69) and median BMI was 29.3 kg/m^2^ (range 18.7–38.1). Successful intraoperative ureter visualization occurred in 2/3, 5/6, and 3/3 participants who received pudexacianinium 0.3 mg, 1.0 mg, or 3.0 mg, respectively. Median intensity values per surgeon assessment were 1 (mild) with the 0.3-mg dose, 2 (moderate) with the 1.0-mg dose, and 3 (strong) with the 3.0-mg dose. A correlation was observed between qualitative (surgeon’s recognition/identification of the ureter during surgery) and quantitative (video recordings of the surgeries after study completion) assessment of fluorescence intensity. Two participants experienced serious adverse events, none of which were drug-related toxicities. One adverse event (grade 1 proteinuria) was related to pudexacianinium. Plasma pudexacianinium concentrations were dose-dependent and the mean (± SD) percent excreted into urine during surgery was 22.3% ± 8.0% (0.3-mg dose), 15.6% ± 10.0% (1.0-mg dose), and 39.5% ± 12.4% (3.0-mg dose).

**Conclusions:**

In this study, 1.0 and 3.0 mg pudexacianinium provided ureteral visualization for the duration of minimally invasive, laparoscopic colorectal procedures and was safe and well tolerated.

**Supplementary Information:**

The online version contains supplementary material available at 10.1007/s00464-023-10193-9.

Intraoperative ureteral injury (IUI) is a rare but serious complication that can occur during abdominal or pelvic surgery [1]. Results of a 10-year (2001–2010) retrospective analysis of ureteral injury in colorectal surgery showed that 6027 (0.28%) cases had been identified in approximately 2 million procedures performed in the United States [2]. More recent results of a retrospective cohort study of approximately 2.5 million abdominopelvic surgeries in the United States (2015–2019) showed that rates of IUI varied by surgery type and were higher than previously reported [3]. Overall, IUIs occurred in 0.88% (95% confidence interval [CI] 0.86–0.90) of gastrointestinal surgeries, 0.29% (95% CI 0.28–0.30) of gynecological surgeries, and 1.17% (95% CI 1.08–1.26) of other abdominopelvic surgeries. Increased risk of IUI was associated with use of preoperative ureteral catheterization or stenting and intraoperative diagnostic cystoscopy. IUIs are associated with higher mortality, morbidity, longer length of hospital stay, increased hospital costs, and specific postoperative complications such as acute renal failure, urinary tract infection, wound complications, and anastomotic leak [2, 4]. Results of a registry study showed that IUI occurred in 1.2% (643 of 53,096) of hysterectomies. In 10% of these cases, the patient lost a kidney because of IUI [5].

Identifying the ureters during surgery can be challenging, especially in patients with complicated diverticulitis, endometriosis, colorectal cancer, gynecologic malignancies, previous surgery, or radiotherapy; therefore, most IUIs are not detected intraoperatively [4, 6]. The risk of IUI may be increased during laparoscopic procedures based on the increased difficulty of detecting the ureter by visual inspection and lack of palpation [7]. Preoperative ureteral stenting has long been proposed as a technique to identify the ureters. However, this is an invasive procedure with associated complications, and the reduction in IUI rate is not significant [8–10]. Other preoperative imaging techniques such as intravenous (IV) or retrograde pyelography and urologic computed tomography can be used to help avoid injury, however these techniques do not provide real-time guidance during surgery [11].

Image-guided surgery using near-infrared fluorescence (NIR-F) imaging technology offers real-time visual information for surgeons and potentially increases patient safety during operations [7]. Methylene blue and indocyanine green (ICG) are commercially available fluorescent dyes, but they are not approved by the US Food and Drug Administration (FDA) for ureter visualization. In addition, the excitation wavelength required by methylene blue limits penetration depth for identifying ureters [12], and ICG is minimally excreted into urine and thus is not suitable for ureteral imaging after IV administration [13].

Pudexacianinium (ASP5354) chloride is an ICG derivative with hydrophilic properties and rapid urinary clearance without metabolism after IV administration designed to enable accurate, real-time ureter visualization during surgery [14]. Photo-optically, pudexacianinium shows NIR-F properties similar to ICG with an absorption peak at 780 nm and a fluorescence emission peak at 820 nm, and is excreted into the urine through the kidneys after IV administration due to its hydrophilicity [15]. In a phase 1, double-blind, randomized, placebo-controlled study, single IV administration of pudexacianinium up to 24 mg was considered safe and well tolerated, urinary excretion was rapid and nearly complete, and 77–100% pudexacianinium was excreted unchanged [15]. Based on these properties, the detection of pudexacianinium using NIR-F imaging technology during excretory flow from the kidneys to the bladder has the potential to facilitate accurate and continuous visualization of the ureter during surgery. The present study was a pudexacianinium dose-finding study for ureter visualization in individuals undergoing laparoscopic, minimally invasive colorectal surgery.

## Materials and methods

### Study design and participants

This was a phase 2, randomized, open-label, dose-ranging clinical trial in adult participants undergoing laparoscopic, minimally invasive colorectal surgery in which the surgeon expected to encounter the ureter in the operative field. This study began on October 15, 2020 at two sites in the United States in accordance with the International Council for Harmonisation guidelines, applicable regulations and guidelines governing study conduct, and the ethical principles with their origin in the Declaration of Helsinki. An institutional review board/independent ethics committee approved the ethical, scientific, and medical appropriateness of the study and reviewed the protocol (and any amendments), written informed consent, and all other documentation prior to study initiation.

Participants were randomly assigned by an interactive response technology system to receive a single dose of pudexacianinium (0.3 mg, 1.0 mg, or 3.0 mg) administered as an IV injection. The investigator defined the left or right ureter as the index ureter to be evaluated for anatomical visualization with a Stryker 1688 Advanced Imaging Modalities 4 K Platform (Stryker Corporation, Kalamazoo, MI) during surgery. Dose ranges were selected using pharmacokinetic (PK) modeling and simulation to provide differentiation around a central dose of 1.0 mg based on both nonclinical findings showing that a 0.01 mg/kg dose provided sufficient ureter visualization 3 h after IV administration [14] and on PK and tolerability results in healthy volunteers [15].

Investigator(s) and the sponsor’s medical and safety representatives met after three participants completed the surgical procedures at each dose level to determine whether to expand a dose under evaluation (up to 15 participants) or discontinue a dose under evaluation. The decision to expand or discontinue a dose was based on the totality of the collected data, including, but not limited to, anatomical visualization (defined as positive visualization of the ureter at both 30 min after dose administration and at the end of surgery) or fluorescence intensity based on a Likert scale (0 = none, 1 = mild, 2 = moderate, and 3 = strong).

Eligible participants were aged ≥ 18 years and scheduled to undergo laparoscopic, minimally invasive colorectal surgery during which ureters needed to be visualized. Participants were required to have adequate liver, renal, and bone marrow function. Exclusion criteria included a history of known retroperitoneal fibrosis, active urinary tract infection, previous exposure to pudexacianinium, and hypersensitivity to pudexacianinium or ICG. Participants were not permitted to have received diuretics, renal transporter inhibitors, or other NIR-F imaging agents, or to have consumed alcohol within 48 h of the study. Additional exclusion criteria included moderate to severe cardiac disease (New York Heart Association Class III-IV), mean resting heart rate of ≤ 45 or ≥ 115 bpm, mean systolic or diastolic blood pressure of ≥ 160 mmHg or ≥ 100 mmHg, respectively, mean corrected QT interval using Fridericia’s formula (QTcF) > 430 ms (male) or > 450 ms (female), or if a requirement for ureteral stenting was anticipated during surgery. Participants enrolled after determination of the selected dose were permitted to have a body mass index (BMI) > 25 kg/m^2^ or an estimated glomerular filtration rate (eGFR) of ≥ 15 and < 60 mL/min/1.73 m^2^ to collect information on visualization in individuals with high BMI and/or renal impairment. All participants provided written informed consent.

### Objectives and endpoints

The primary objective was to select the dose of pudexacianinium for ureter visualization in participants undergoing laparoscopic/minimally invasive colorectal surgery. The primary efficacy endpoint was successful anatomical visualization of the index ureter, defined as answering “yes” to the question “Can the ureter be adequately visualized with NIR-F?” at both 30 min after dosing and at the end of surgery. If the answer was “no” at either time point, visualization was deemed unsuccessful for that participant.

Secondary objectives were to investigate the safety, tolerability, and PK of pudexacianinium; corresponding secondary endpoints included adverse events (AEs), 12-lead electrocardiogram data, pudexacianinium plasma concentrations, and urinary pudexacianinium concentration during surgery. Exploratory objectives were to investigate the relationship between the PK and pharmacodynamics of pudexacianinium, the fluorescence intensity of pudexacianinium, the duration of ureter visualization, and the benefit of visualization using pudexacianinium during surgery. Exploratory endpoints were visualization of the contralateral ureter when amenable to visualization and qualitative assessments (using a 4-point Likert scale) of fluorescence intensity and signal-to-background ratio.

### Assessments

Efficacy endpoints were based on intraoperative ureter fluorescence visualization of the surgical field elicited using an NIR-F imaging system (FDA 510[k] cleared Stryker 1688 AIM system) positioned to visualize the index ureter. After pudexacianinium was administered, surgery was initiated and ureter visualization was assessed at time points of 10, 20, 30, 45, and 60 min, then every 30 min thereafter, and/or until the last time point before removal of visualization instruments at the end of surgery. At the investigator’s discretion, the contralateral ureter could be visualized at similar time points when feasible. Video images were captured in bright-field (white light), NIR-F, and overlay modes and recorded during the entire surgery. Anatomical visualization of the index ureter was assessed intraoperatively by the investigator as “yes” or “no” on the ability to visualize the ureter at each time point. Qualitative assessment of fluorescence intensity was performed using a Likert scale (0=none, 1=mild, 2=moderate, and 3=strong).

Adverse events were recorded and graded using the National Cancer Institute Common Terminology Criteria for Adverse Events, version 5.0. Causal relationships between AEs and pudexacianinium were determined as assessed by the investigator. Safety assessments also included clinical laboratory evaluations (hematology, serum chemistry, and urinalysis), 12-lead electrocardiogram, vital signs measurements, and physical examination. Change from baseline in QTcF was determined for each participant using the mean value of triplicate readings at each time point.

For the first three participants, blood samples for PK assessments were collected prior to pudexacianinium administration, at 10, 30, and 60 min after administration, then every 30 min thereafter until a final collection was obtained at the end of surgery (or at 180 min if the surgery was completed in less than 105 min). For the subsequent nine patients enrolled after the protocol was amended, blood was collected prior to administration, then at 10 min and either 30 or 60 min after administration, and at the end of surgery (or at 180 min if the duration of surgery was less than 105 min). Total urine samples to assess the amount of pudexacianinium excreted in the urine were collected during surgery. Before protocol amendment, point urine samples for PK assessments were collected prior to pudexacianinium administration, at 10, 30, and 60 min after administration, then every 30 min thereafter until a final collection was obtained at the end of surgery. Urine sample collection at all time points was not feasible during certain portions of surgical procedures. After protocol amendment, point urine samples were collected pre-dose and at the end of surgery.

Fluorescence intensity was qualitatively assessed as dye visibility by the investigator at each time point using the 4-point Likert scale. The duration of visualization of the index ureter(s) was based on the result of the first and the last positive visualized time point. Investigators were asked to assess the clinical benefit of visualization provided by pudexacianinium during surgery by answering “yes” or “no” to the following questions:

- Was the location of the index ureter(s) as expected?
- Did visualization of the index ureter(s) occur with white light at 30 min after pudexacianinium administration?
- Did visualization of the index ureter(s) occur with white light at the end of surgery?
- Was NIR-F superior to white light in terms of visualization of the index ureter(s)?
- Did the location of the ureter visualized by NIR-F alter the operative plan?
- Were any ureter anomalies or abnormalities observed?

The contralateral ureter was assessed using the same binary questions for anatomical visualization and Likert scale when feasible.

A post hoc analysis (not protocol-specified) was conducted to quantitatively assess fluorescence intensity. Full color images of ureters taken during surgery were decomposed into red (R), green (G), and blue (B) components using ImageJ video analysis software (https://imagej.nih.gov/ij/). As the fluorescence emitted by pudexacianinium is displayed as green in color in the surgical video, the color contrast between the dye fluorescence in the ureter and the surrounding tissues was quantified by calculating a ratio of the signal levels G/(R + B) where R, G, and B are the average values of the signal for each color. A commensurate ratio value was calculated for signals emanating from the tissues surrounding the ureter lumen. A comparison of these two ratio values was expressed as a “contrast enhancement factor” (CEF), where CEF = (G/(R + B)_inside_)/(G/(R + B)_outside_), to quantify the color contrast that the surgeon observes when pudexacianinium is present in the ureter and is visualized with a Stryker camera and light source. A CEF value was calculated for the case when dye was present in the ureter (“dye”) and for the case when no dye was present (“without dye”). Fluorescence intensity was qualitatively assessed as dye visibility by the surgeon at each time point using the 4-point Likert scale.

### Statistical analysis

Sample size was selected to provide adequate information to determine the dose of pudexacianinium for ureter visualization and was not determined based on a statistical power calculation.

Efficacy analyses were performed based on the full analysis set, defined as all participants who received pudexacianinium and had at least one ureter visualization assessment during surgery. Safety was evaluated for the safety analysis set, defined as all participants who received pudexacianinium. Pharmacokinetic assessments were performed on the PK analysis set, defined as all participants who received at least one dose of pudexacianinium and had at least one plasma or urine concentration data point available at the time of dosing and sampling.

All data were summarized descriptively using mean, SD, minimum, median, and maximum for continuous variables, and frequency and percentage for categorical data. No hypothesis testing or statistical comparisons were performed.

## Results

### Participants

Of 19 individuals who enrolled, six were excluded at screening and one did not receive pudexacianinium (Supplemental Fig. 1). The 12 remaining participants received 0.3 mg (*n* = 3), 1.0 mg (*n* = 6), or 3.0 mg (*n* = 3) pudexacianinium and were included in the analysis. After the first nine participants (three participants at each dose) had completed the study, the study investigators and sponsor decided to enroll three additional participants at the 1.0-mg dose of pudexacianinium to further assess ureter visualization. All three participants had BMI ≥ 25 kg/m^2^. No participants with eGFR ≥ 15 and < 60 mL/min/1.73 m^2^ were enrolled in the study.

In the total study population (*n* = 12), most participants were female (*n* = 10; 83.3%) and most were White (*n* = 10; 83.3%); median age was 54 years (range, 24–69 years) and median BMI was 29.3 kg/m^2^ (range 18.7–38.1) (Table 1). Surgical procedures included total colectomy (*n* = 2), right hemicolectomy (*n* = 4), proctocolectomy (*n* = 1), lower anterior resection (*n* = 1), sigmoid resection/lower anterior resection (*n* = 2), rectosigmoid/lower anterior resection (*n* = 1), and appendectomy (*n* = 1). Surgeries ranged in duration from 59 to 186 min.

**Table 1:** Participant demographics

	Pudexacianinium			Total (*N* = 12)
	0.3 mg (*n* = 3)	1.0 mg (*n* = 6)	3.0 mg (*n* = 3)	
Age, years, median (min, max)	55 (53, 58)	57 (46, 69)	45 (24, 46)	54 (24, 69)
Female, *n* (%)	3 (100)	5 (83.3)	2 (66.7)	10 (83.3)
Race, *n* (%)				
White	2 (66.7)	5 (83.3)	3 (100)	10 (83.3)
Black or African American	1 (33.3)	0	0	1 (8.3)
Asian	0	1 (16.7)	0	1 (8.3)
Ethnicity, *n* (%)				
Hispanic or Latino, *n* (%)	1 (33.3)	2 (33.3)	1 (33.3)	4 (33.3)
Weight, mean (SD) kg	68.2 (21.8)	83.7 (11.9)	68.4 (20.4)	76.0 (17.1)
BMI, kg/m^2^				
Mean (SD) kg/m^2^	26.7 (10.0)	29.4 (3.7)	26.8 (7.1)	28.1 (6.0)
Median	22.6	29.5	29.5	29.3
Min, max	19.4, 38.1	25.1, 34.3	18.7, 32.1	18.7, 38.1

*BMI* body mass index, *SD* standard deviation

### Efficacy

**Fig. 1:**
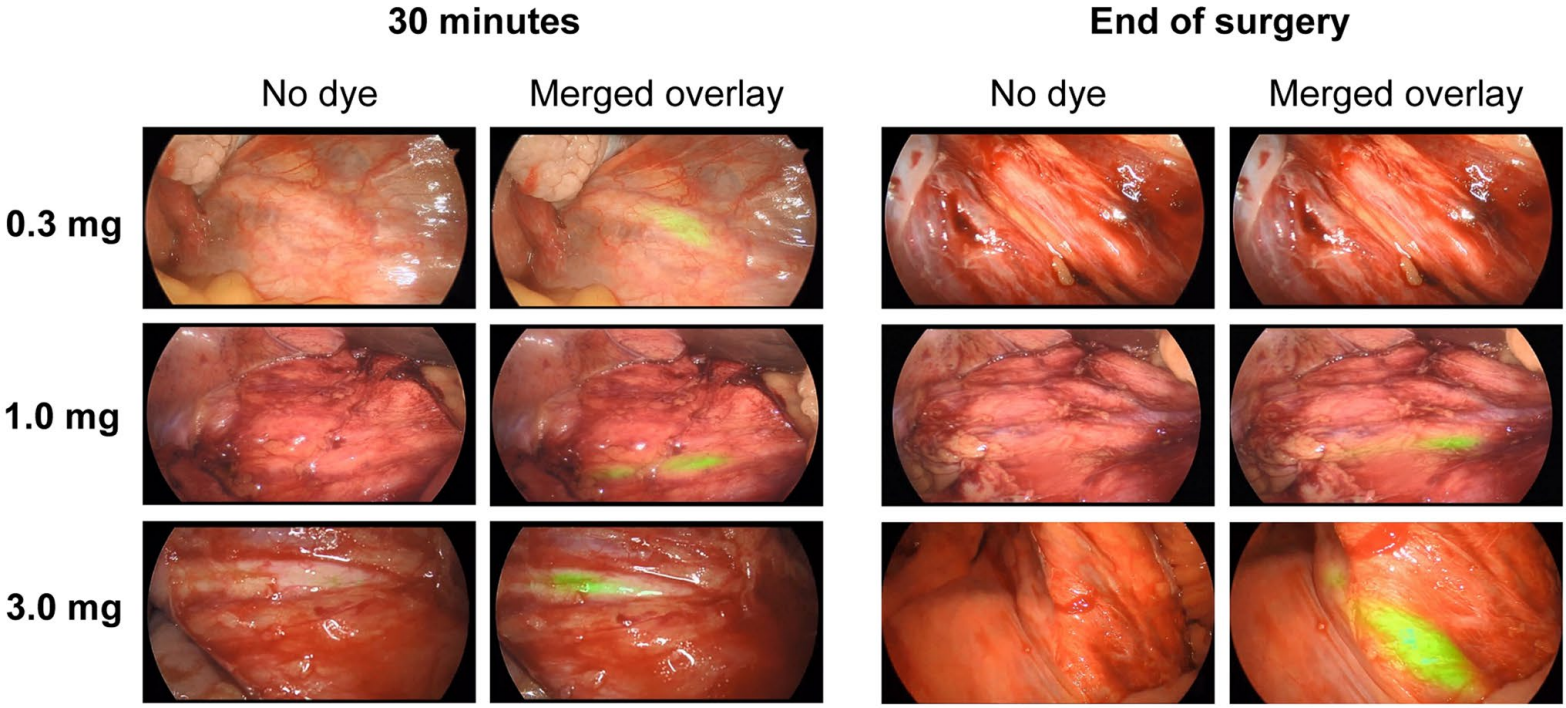
Ureter visualizations for individual participants by dose at 30 min after pudexacianinium administration and the end of surgery

**Fig. 2:**
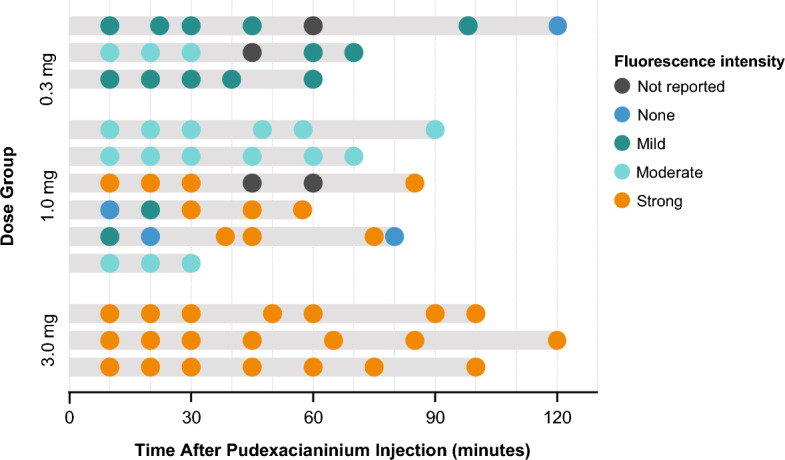
Fluorescence intensity of the index ureter at each time point during surgery

**Fig. 3:**
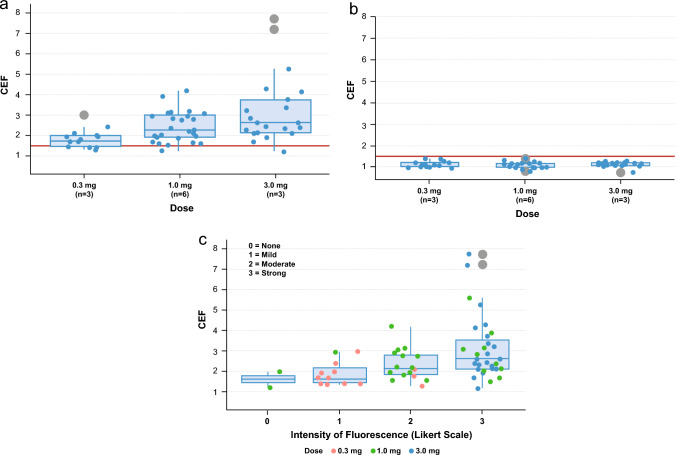
Distribution of CEF **a** with dye, or **b** without dye. **c** Correlation between the qualitative and quantitative assessment of fluorescent intensity. This figure shows a correlation between the qualitative assessment of fluorescence intensity determined by the surgeon and the quantitative assessment determined using video records by Stryker colleagues. Dots show observations in each dose. The lower and upper hinges correspond to the first and third quartiles (25th and 75th percentiles). The upper whisker extends from upper hinge to 1.5 × IQR. The lower whisker extends from lower hinge to 1.5 × IQR. Grey dots show outliers (data beyond the end of the whiskers). The red line illustrates the CEF threshold (1.5). CEF, contrast enhancement factor; IQR, interquartile range

**Fig. 4:**
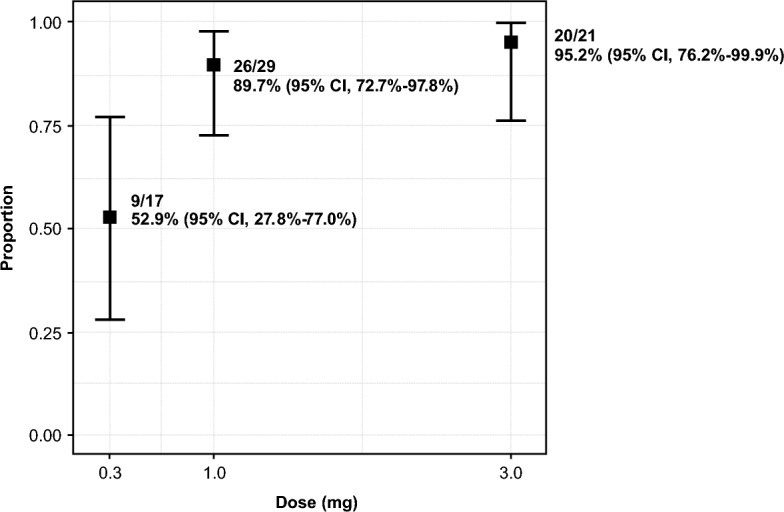
Proportion of data points at each pudexacianinium dose with a CEF above threshold (1.5). The figure shows the number of observations above CEF ˃1.5/total number of observations. Proportions and 95% CIs are presented. CEF, contrast enhancement factor; CI, confidence interval

All three doses of pudexacianinium provided enhanced anatomical visualization of ureters. Sample images of visualization during surgery from each dose group are presented in Fig. 1. In the primary endpoint analysis, successful visualization of the index ureter at both 30 min after dosing and at the end of surgery was observed in 2/3 (66.7%), 5/6 (83.3%), and 3/3 (100%) participants in the 0.3-mg, 1.0-mg, and 3.0-mg dose groups, respectively (Table 2). The median fluorescence intensity values based on investigator assessment were 1 (mild) in the 0.3-mg dose group, 2 (moderate) in the 1.0-mg dose group, and 3 (strong) in the 3.0-mg dose group (Fig. 2). The median (range) CEF factor with dye and without dye was 1.77 (1.3–3.0) and 1.05 (0.9–1.3), respectively, for the 0.3-mg dose, 2.28 (1.3–5.6) and 1.06 (0.8–1.4), respectively, for the 1.0-mg dose, and 2.64 (1.2–7.7) and 1.10 (0.7–1.2), respectively, for the 3.0-mg dose (Fig. 3a, b and Table 3). All data points under “without dye” conditions showed CEF values of approximately 1.0. As CEF values were typically greater than 1.5 with dye, the threshold for dye identification was set at this value. A correlation was observed between the surgeon’s qualitative assessment of fluorescence intensity (the surgeon’s ability to recognize/identify the ureter during surgery) and the quantitative assessment of fluorescence intensity (assessed using video recordings of the surgeries after study completion) (Fig. 3c). Most data points of ureter visualization for patients in the 1.0-mg (89.7%) and 3.0-mg (95.2%) dose groups had CEF values above the threshold (Fig. 4).

**Table 2:** Index and contralateral ureter visualization from 10 min after pudexacianinium administration through the end of the surgical procedure

*n*/*N*^a^ (%)	Index ureter			Contralateral ureter		
	0.3 mg (*n* = 3)	1.0 mg (*n* = 6)	3.0 mg (*n* = 3)	0.3 mg (*n* = 3)	1.0 mg (*n* = 6)	3.0 mg (*n* = 3)
Successful visualization^b^	2/3 (66.7)	5/6 (83.3)	3/3 (100)	2/3 (66.7)	2/4 (50.0)	1/2^c^ (50.0)
Positive visualization by time point						
10 min	3/3 (100)	5/6 (83.3)	3/3 (100)	2/2 (100)	1/3 (33.3)	1/1 (100)
20 min	3/3 (100)	5/6 (83.3)	3/3 (100)	2/2 (100)	2/2 (100)	1/1 (100)
30 min	3/3 (100)	5/5 (100)	3/3 (100)	2/2 (100)	2/3 (66.7)	1/1 (100)
45 min	2/2 (100)	4/4 (100)	3/3 (100)	1/1 (100)	3/3 (100)	1/1 (100)
60 min	1/1 (100)	2/2 (100)	3/3 (100)	2/2 (100)	2/2 (100)	2/2 (100)
90 min	1/1 (100)	1/1 (100)	3/3 (100)	1/1 (100)	0/0	2/2 (100)
End of surgery	2/3 (66.7)	5/6 (83.3)	3/3 (100)	2/3 (66.7)	4/4 (100)	2/2 (100)

^a^N is the number of participants with nonmissing anatomical visualization assessments at the respective analysis time point. Missing values were considered visualization failures

^b^Defined as positive anatomical visualization of the index ureter both 30 min after pudexacianinium dosing and at the end of surgery. Percentages are based on the nonmissing assessments at the respective time point and were counted as nonsuccess if values were missing at either time point

^c^One patient was counted as having “nonsuccessful visualization” because of the missing assessment for the contralateral ureter at the 30-min time point

**Table 3:** Detailed index ureter visualization and quantitative results

Pudexacianinium dose	Participants			Surgery type	Index ureter	Duration of surgery (minutes)	Duration of visualization (minutes)^a^	Ureter visualized		Intensity score^b^		Mean (SD)/Median (Range) CEF^c^	
	Sex	Age (y)	BMI (kg/m^2^)					30 min	End	30 min	End	With dye	Without dye
0.3 mg	F	53	22.6	Total colectomy	R	147	89	Yes	No	1	0	1.84 (0.46)/1.77 (1.3–3.0)	1.10 (0.13)/1.05 (0.9–1.3)
	F	58	19.4	Hemicolectomy (R)	R	92	61	Yes	Yes	2	1		
	F	55	38.1	Hemicolectomy (R)	R	112	52	Yes	Yes	1	1		
1.0 mg	F	55	29.9	Lower anterior resection	L	134	85	Yes	Yes	2	2	2.55 (0.95)/2.28 (1.3–5.6)	1.06 (0.12)/1.06 (0.8–1.4)
	M	46	25.6	Sigmoid resection/lower anterior resection	L	110	64	Yes	Yes	2	2		
	F	69	32.5	Hemicolectomy (R)	R	139	76	Yes	Yes	3	3		
	F	62	29.1	Sigmoid resection/lower anterior resection	L	154	44	Yes	Yes	3	3		
	F	59	25.1	Rectosigmoid/lower anterior resection	L	122	66	Yes	No	3	0		
	F	51	34.3	Appendectomy	R	59	23	Yes^d^	Yes	ND^e^	2		
3.0 mg	F	46	32.1	Hemicolectomy (R)	R	130	91	Yes	Yes	3	3	3.23 (1.70)/2.64 (1.2–7.7)	1.09 (0.10)/1.10 (0.7–1.2)
	M	45	29.5	Proctocolectomy	L	186	112	Yes	Yes	3	3		
	F	24	18.7	Total colectomy	R	185	92	Yes	Yes	3	3		

*BMI* body mass index, *CEF* contrast enhancement factor, *F* female, *L* left, *M* male, *ND* not done, *R* right, *SD* standard deviation

^a^Duration of visualization is defined as the last actual time of visualization minus the first actual time of visualization plus 1 min

^b^0 = none; 1 = mild; 2 = moderate; 3 = strong

^c^As some color contrast between the ureter and the surrounding tissue may exist naturally, the degree to which this contrast is enhanced by the presence of the dye in the ureter may be quantified by additionally calculating a ratio defined as CEF = (G/(R + B)_inside_)/(G/(R + B)_outside_)

^d^Ureter visualization at 30 min is an imputed value as the 30 min time point also represents the end of surgery time point

^e^Intensity at the 30 min time point was not performed as the 30 min time point also represents the end of surgery time point

In the exploratory analysis of the benefits of visualization by pudexacianinium during surgery, consensus was achieved on all questions, suggesting clinical benefit in both the 1.0-mg and 3.0-mg dose groups (Table 4). Of note, according to investigators, visualization of the index ureter at the end of surgery in the 0.3-mg dose group was not possible with white light in two (66.7%) participants and NIR-F was not considered superior to white light for visualizing the index ureter in one (33.3%) participant (Table 4).

**Table 4:** Clinical benefits of visualization by pudexacianinium during surgery

*n* answering “yes,” (%)	Pudexacianinium		
	0.3 mg (*n* = 3)	1.0 mg (*n* = 6)	3.0 mg (*n* = 3)
The location of the index ureter was as expected	3 (100)	6 (100)	3 (100)
Visualization of the index ureter occurred with white light 30 min after pudexacianinium administration	3 (100)	6 (100)	3 (100)
Visualization of the index ureter occurred with white light at the end of surgery	1 (33.3)	6 (100)	3 (100)
NIR-F was superior to white light in terms of visualization of the index ureter	2 (66.7)	6 (100)	3 (100)
The location of the ureter visualized by NIR-F did not alter the operative plan	3 (100)	6 (100)	3 (100)
No ureter anomalies or abnormalities were observed	3 (100)	6 (100)	3 (100)

*NIR-F* near-infrared fluorescence

### Safety

A total of six participants experienced AEs, one of which was considered related to treatment (proteinuria grade 1, 1.0-mg dose group, resolved after 11 days, based on results of repeated urinalysis) (Table 5). The participant with grade 1 treatment-related proteinuria had baseline, postoperative, and follow-up creatinine levels of 79.6, 88.4, and 79.6 µmol/L, respectively; eGFR was ≥ 60 mL/min/1.73m^2^ at each time point. Two (22.2%) participants experienced serious AEs, one of whom experienced perforation of the rectal stump for which histology was positive for colon cancer (coded as rectal perforation and colon cancer). The other participant experienced blood loss anemia. No serious AEs were considered drug related, and no deaths occurred during the study.

**Table 5:** Safety summary

*n* (%)	Pudexacianinium			
	0.3 mg (*n* = 3)	1.0 mg (*n* = 6)	3.0 mg (*n* = 3)	Total (*N* = 12)
AE	2 (66.7)	5 (50.0)	1 (33.3)	6 (50.0)
Drug-related AE	0	1 (16.7)	0	1 (8.3)
Serious AE	2 (66.7)	0	0	2 (16.7)
Worst grade ≥ 3 AE	2 (66.7)	0	0	2 (16.7)
Deaths	0	0	0	0
AEs by organ class				
Blood and lymphatic system disorders, *n* (%)	1 (33.3)	0	0	1 (8.3)
Blood loss anemia^a^	1 (33.3)	0	0	1 (8.3)
Gastrointestinal disorders, *n* (%)	1 (33.3)	0	1 (33.3)	2 (16.7)
Abdominal rigidity	0	0	1 (33.3)	1 (8.3)
Rectal perforation^a^	1 (33.3)	0	0	1 (8.3)
Injury, poisoning, procedural complications, *n* (%)	0	2 (33.3)	0	2 (16.7)
Incision site erythema	0	1 (16.7)	0	1 (8.3)
Procedural nausea	0	1 (16.7)	0	1 (8.3)
Procedural pain	0	1 (16.7)	0	1 (8.3)
Musculoskeletal and connective tissue disorders, *n* (%)	1 (33.3)	0	0	1 (8.3)
Muscle spasms	1 (33.3)	0	0	1 (8.3)
Neoplasms benign, malignant, and unspecified, *n* (%)	1 (33.3)	0	0	1 (8.3)
Colon cancer^a^	1 (33.3)	0	0	1 (8.3)
Renal and urinary disorders				
Hematuria	0	1 (16.7)	0	1 (8.3)
Proteinuria	0	1 (16.7)	0	1 (8.3)
Drug-related AE				
Proteinuria	0	1 (16.7)	0	1 (8.3)

*AE* adverse event

^a^Serious AE unrelated to treatment

Changes from baseline in QTcF were observed in all participants during the postoperative period. Change was between 30 and 60 ms for all participants (*n* = 3) who received the 0.3-mg dose and ≤ 30 ms for all participants (*n* = 3) who received the 3.0-mg dose; change was between 30 and 60 ms for half (*n* = 3) and ≤ 30 ms for half (*n* = 3) of participants who received the 1.0-mg dose (Table 6). During the follow-up period, QTcF values reverted back to baseline (change ≤ 0 ms) for eight (66.7%) participants, while the remaining four (33.3%) participants experienced changes of ≤ 30 ms.

**Table 6:** Change from baseline in QTcF by analysis visit

*n* (%)	Pudexacianinium			
	0.3 mg (*n* = 3)	1.0 mg (*n* = 6)	3.0 mg (*n* = 3)	Total (*N* = 12)
Postoperative				
≤ 0 ms	0	0	0	0
> 0 to ≤ 30 ms	0	3 (50.0)	3 (100)	6 (50.0)
> 30 to ≤ 60 ms	3 (100)	3 (50.0)	0	6 (50.0)
> 60 ms	0	0	0	0
Follow-up				
≤ 0 ms	1 (33.3)	5 (83.3)	2 (66.7)	8 (66.7)
> 0 to ≤ 30 ms	2 (66.7)	1 (16.7)	1 (33.3)	4 (33.3)
> 30 to ≤ 60 ms	0	0	0	0
> 60 ms	0	0	0	0
Any postbaseline time point				
≤ 0 ms	0	0	0	0
> 0 to ≤ 30 ms	0	3 (50.0)	3 (100.0)	6 (50.0)
> 30 to ≤ 60 ms	3 (100)	3 (50.0)	0	6 (50.0)
> 60 ms	0	0	0	0

*QTcF* QT interval with Fridericia’s correction

### Pharmacokinetics

Plasma pudexacianinium concentrations were dose-dependent, but slightly less than dose-proportional (Table 7). The mean (± SD) percentage of drug dose excreted into urine during surgery was 22.3% ± 8.0% with the 0.3-mg dose, 15.6% ± 10.0% with the 1.0-mg dose, and 39.5% ± 12.4% with the 3.0-mg dose of pudexacianinium.

**Table 7:** Pudexacianinium plasma concentration over time and total urine excretion during surgery

Time	0.3 mg (*n* = 3)	1.0 mg (*n* = 6)	3.0 mg (*n* = 3)
Plasma concentration, ng/mL, mean (SD)			
Predose	0 (0)	0 (0)	0 (0)
10 min	48.9 (20.6)	234 (207)	322 (177)
30 min	22.5 (3.3)	67.2 (15.5)	146 (46.1)
60 min	15.3 (N/A)^a^	38.2 (7.4)^b^	106 (N/A)^a^
90 min	12.7 (N/A)^a^	34.4 (N/A)^a^	NR
120 min	8.4 (N/A)^a^	NR	NR
End of surgery	14.0 (6.0)	39.5 (15.7)	47.3 (29.8)^b^
180 min	NR	29.8 (16.6)^a^	NR
Pudexacianinium excreted in urine, mean (SD)			
mg	0.067 (0.024)	0.156 (0.100)	1.19 (0.37)
%	22.3 (8.0)	15.6 (10.0)	39.5 (12.4)
Urine collection interval, min			
Mean (SD)	113.0 (27.0)	87.0 (27.0)	142.0 (35.0)
Median (range)	106 (91–143)	93 (30–115)	157 (102–168)

*N/A* not applicable, *NR* not reported (*n* = 0), *SD* standard deviation

^a^*n* = 1

^b^*n* = 2

## Discussion

Results of this phase 2, dose-ranging study show that IV administration of pudexacianinium leads to enhanced anatomical visualization of the ureter under NIR-F conditions at all doses. Sustained enhancement of ureter visualization was observed for five of the six patients who received the 1.0-mg dose and all three patients who received the 3.0-mg dose during surgery. According to the visualization data and PK findings, plasma pudexacianinium concentrations and urinary pudexacianinium excretion increase in a dose-dependent manner. Results of the quantitative assessment of color contrast showed that the CEF was notably less for the 0.3-mg dose than for the 1.0-mg or 3.0-mg dose of pudexacianinium, indicating that the higher doses provide greater assurance of enabling the surgeon to visualize the ureter consistently. Ureters were visualized at the end of surgery for all except two participants (one received the 0.3-mg dose and one received the 1.0-mg dose). It is important to note that BMI is an important factor to consider when assessing optical visualization of vessels beneath the tissue surface. In the current study, participants who received pudexacianinium 1.0 mg had higher mean and median BMIs than participants in the other dose cohorts.

Pudexacianinium appeared to be safe and well tolerated in this study. One case of proteinuria grade 1 was considered related to pudexacianinium (1.0-mg dose) administration, and the few serious AEs that occurred were not related to pudexacianinium and consistent with the underlying indication of colorectal surgery. It is also important to note that no AEs observed were suggestive of hypersensitivity or anaphylactic reactions, which have been reported in isolated cases immediately following administration of high doses of ICG [16–19]. The root cause of these reactions on ICG is yet to be determined but may be related to the iodine component, which is not present in pudexacianinium. No hypersensitivity reactions were reported for any participants in the completed phase 1 trial [15], nor were any toxicity reactions observed at potential target doses in preclinical studies [14]. Any potential risk is further mitigated by the administration of pudexacianinium solely within the confines of a surgical suite/operating room, an environment well-equipped to monitor and treat hypersensitivity reactions in the unlikely event they might occur.

Published rates of IUI varied by surgery type and may be higher than previously reported [3]. Image-guided surgery using NIR-F imaging is a promising technique that offers real-time visualization for surgeons, helping minimize the risk for IUI and potentially leading to better overall patient outcomes. Because no imaging agents are yet approved by the FDA for ureter visualization, current options are limited to off-label use of methylene blue and ICG, or stents, which have been associated with increased time in the operating room, medical costs, and potential for ureteral injury [8, 9]. Results from the nonclinical pharmacology in vivo models, both in laparoscopic and open surgeries, were predictive of the enhanced ureter visualization observed in adult patients undergoing laparoscopic/minimally invasive colorectal surgery [14].

The 1.0-mg pudexacianinium dose provided visualization throughout the duration of surgery for five of the six participants. The surgeon survey on clinical benefit showed that benefits of visualization (6/6 questions) were apparent in all participants at the 1.0 mg and 3.0 mg doses. By comparison, the 0.3-mg dose provided reduced fluorescence intensity and shorter duration of visualization that was considered inadequate for intraoperative ureter visualization, while the 3.0-mg dose provided stronger fluorescence intensity over the 1.0-mg dose.

Taken together with previous findings to date on pudexacianinium, we anticipate that this imaging agent will provide clinical benefit by addressing the unmet medical need for improved ureter identification using a safe, noninvasive, intraoperative visualization agent. Moreover, the benefits of avoiding intraoperative stents should minimize ureteral injuries and associated complications, potentially decrease operating times, avoid repeat surgeries, and reduce the possibility of increased medical costs from side effects.

This study was limited by the small sample size with descriptive statistics used to evaluate differences between doses. Additionally, no participants with renal impairment were included in the study, so it remains to be determined whether slower renal elimination might impact fluorescence intensity and duration of ureter visualization. An investigation of the safety and tolerability and of the PK parameters of pudexacianinium in individuals with renal impairment is currently planned (NCT05495581).

In conclusion, this phase 2 study demonstrated that administration of the novel fluorescent imaging agent pudexacianinium, at doses of 1.0 mg and 3.0 mg, provides ureteral visualization for the full duration of minimally invasive, laparoscopic colorectal procedures. The CEF threshold of 1.5 for dye identification is relevant based on ureter identification and visualization that occurred at CEF values above the threshold. Both qualitatively and quantitatively, data indicated that there was a direct relationship between signal intensity and pudexacianinium dose. High proportions of patients in the 1.0-mg (89.7%) and 3.0-mg (95.2%) dose groups had CEF values above 1.5, with potential benefit of the 3.0-mg versus 1.0-mg dose based on the higher proportion of patients above the dye identification threshold. Pudexacianinium, across studies to date, has demonstrated a consistent and desirable safety, tolerability, and PK profile. In the current study, all doses of pudexacianinium were tolerable and had good safety profiles.

## Supplementary Information

Below is the link to the electronic supplementary material.

Supplementary file1 (MP4 27158 KB)

Supplementary file2 (DOCX 39 KB)

## Data Availability

Researchers may request access to anonymized participant level data, trial level data and protocols from Astellas sponsored clinical trials at www.clinicalstudydatarequest.com. For the Astellas criteria on data sharing see: https://clinicalstudydatarequest.com/Study-Sponsors/Study-Sponsors-Astellas.aspx.

## References

[1] Yellinek S, Krizzuk D, Nogueras JJ, Wexner SD (2018) Ureteral injury during colorectal surgery: two case reports and a literature review. J Anus Rectum Colon 2:71–76 10.23922/jarc.2017-052 PMC6752145 31559346

[2] Halabi WJ, Jafari MD, Nguyen VQ, Carmichael JC, Mills S, Pigazzi A, Stamos MJ (2014). Ureteral injuries in colorectal surgery: an analysis of trends, outcomes, and risk factors over a 10-year period in the United States. Dis Colon Rectum.

[3] McCarus S, Alexandre A, Kimura T, Feng Q, Han W, Shortridge E, Lima R, Schwart J, Wexner S (2023) Abdominopelvic surgery: intraoperative ureteral injury and prophylaxis in the United States, 2015–2019. Adv Ther 40:3169–3185 10.1007/s12325-023-02515-z PMC10272259 37227585

[4] Brandes S, Coburn M, Armenakas N, McAninch J (2004). Diagnosis and management of ureteric injury: an evidence-based analysis. BJU Int.

[5] Ravlo M, Moen MH, Bukholm IRK, Lieng M, Vanky E (2022). Ureteric injuries during hysterectomy-A Norwegian retrospective study of occurrence and claims for compensation over an 11-year period. Acta Obstet Gynecol Scand.

[6] Gilmour DT, Dwyer PL, Carey MP (1999). Lower urinary tract injury during gynecologic surgery and its detection by intraoperative cystoscopy. Obstet Gynecol.

[7] Slooter MD, Janssen A, Bemelman WA, Tanis PJ, Hompes R (2019). Currently available and experimental dyes for intraoperative near-infrared fluorescence imaging of the ureters: a systematic review. Tech Coloproctol.

[8] Burks FN, Santucci RA (2014). Management of iatrogenic ureteral injury. Ther Adv Urol.

[9] Feingold D, Steele SR, Lee S, Kaiser A, Boushey R, Buie WD, Rafferty JF (2014). Practice parameters for the treatment of sigmoid diverticulitis. Dis Colon Rectum.

[10] Kitrey ND, Campos-Juanatey F, Hallscheidt P, Serafetinidis E, Sharma DM, Waterloos M (2022) EAU Guidelines on Urological Trauma. European Association of Urology. Available at: https://d56bochluxqnz.cloudfront.net/documents/full-guideline/EAU-Guidelines-on-Urological-Trauma-2022.pdf 10.1016/j.euf.2023.08.011 37968186

[11] Ahn CB, Kim JH, Park GK, Park KY, Bao K, Lee JW, Choi HS, Son KH (2019). Prognostic imaging of iatrogenic and traumatic ureteral injury by near-infrared fluorescence. Quant Imaging Med Surg.

[12] Verbeek FP, van der Vorst JR, Schaafsma BE, Swijnenburg RJ, Gaarenstroom KN, Elzevier HW, van de Velde CJ, Frangioni JV, Vahrmeijer AL (2013). Intraoperative near infrared fluorescence guided identification of the ureters using low dose methylene blue: a first in human experience. J Urol.

[13] Friedman-Levi Y, Larush L, Diana M, Marchegiani F, Marescaux J, Goder N, Lahat G, Klausner J, Eyal S, Magdassi S, Nizri E (2018). Optimization of liposomal indocyanine green for imaging of the urinary pathways and a proof of concept in a pig model. Surg Endosc.

[14] Fushiki H, Yoshikawa T, Matsuda T, Sato T, Suwa A (2021). Preclinical development and validation of ASP5354: a near-infrared fluorescent agent for intraoperative ureter visualization. Mol Imaging Biol.

[15] Murase T, Takizawa M, Galitz L, Flach S, Murray V, Gufford B, Suwa A (2021). Randomized, double-blind, controlled study to evaluate safety and pharmacokinetics of single ascending doses of ASP5354, an investigational imaging product, in healthy adult volunteers. Clin Pharmacol Drug Dev.

[16] Chu W, Chennamsetty A, Toroussian R, Lau C (2017). Anaphylactic shock after intravenous administration of indocyanine green during robotic partial nephrectomy. Urol Case Rep.

[17] Kim M, Lee S, Park JC, Jang DM, Ha SI, Kim JU, Ahn JS, Park W (2020). Anaphylactic shock after indocyanine green video angiography during cerebrovascular surgery. World Neurosurg.

[18] Olsen TW, Lim JI, Capone A, Myles RA, Gilman JP (1996). Anaphylactic shock following indocyanine green angiography. Arch Ophthalmol.

[19] Wolf S, Arend O, Schulte K, Reim M (1992). Severe anaphylactic reaction after indocyanine green fluorescence angiography. Am J Ophthalmol.

