# Supplementary figures and images for: Pudexacianinium (ASP5354) chloride for ureter visualization in participants undergoing laparoscopic, minimally invasive colorectal surgery
Source: Surg Endosc. 2023 Jul 20;37(9):7336–47. doi: 10.1007/s00464-023-10193-9 (PMC10462524; doi:10.1007/s00464-023-10193-9)

**Supplemental Figure 1. Study flow diagram.**


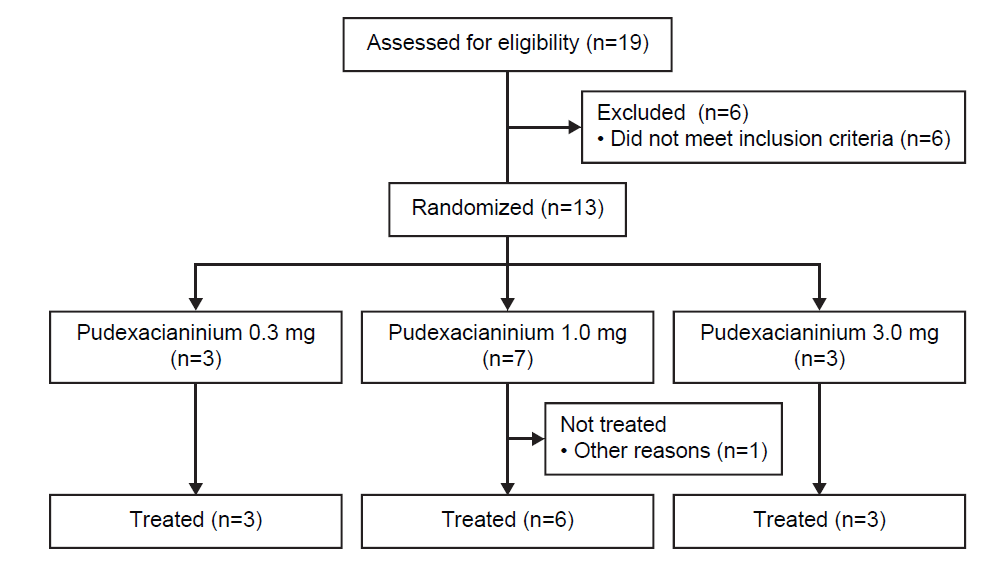

Supplement: Supplementary file 2 — Supplementary file2 (DOCX 39 KB) [file 464_2023_10193_MOESM2_ESM.docx]
